# Impact of exercise training and sleep on children’s stress perception

**DOI:** 10.3389/fspor.2025.1609029

**Published:** 2025-09-23

**Authors:** Eleonora Pagani, Gianluigi Oggionni, Luca Giovanelli, Anna Mariani, Giuseppina Bernardelli, Daniela Lucini

**Affiliations:** ^1^ Department of Psychology, Catholic University of the Sacred Heart, Milan, Italy; ^2^ Exercise Medicine Unit, IRCCS Istituto Auxologico Italiano, Milan, Italy; ^3^ BIOMETRA Department, University of Milan, Milan, Italy; ^4^ DISCCO Department, University of Milan, Milan, Italy

**Keywords:** physical activity, youth, wellbeing, family environments, adolescents, parents, lifestyle

## Abstract

**Background:**

A healthy lifestyle is currently considered a pillar in the prevention/treatment of chronic non-communicable diseases in both adulthood and childhood. Notably, it is also a prominent tool for fostering wellbeing and managing stress, particularly at a young age when these two lifestyle components play a fundamental role in determining present and future health.

**Purpose:**

This study aimed to evaluate the link between stress perception and lifestyle habits, particularly exercise and sleep in children/adolescents and their parents, using a simple anonymous questionnaire

**Methods:**

Members of 50 families filled out a simple *ad hoc* anonymous questionnaire on lifestyle habits (exercise, sleep, nutrition, etc.) and stress/health/performance perceptions. The questionnaire was based on standardized instruments (International Physical Activity Questionnaire and the American Heart Association Healthy Diet Score), including objective indicators such as exercise volume and self-developed ordinal evaluation scales ranging from 0 to 10 that assessed subjective perceptions (e.g., health, stress, and performance). Anthropometric, systolic and diastolic arterial pressures, and heart rate data were also collected.

**Results:**

In children/adolescents, stress perception negatively correlated with the volume of moderate–vigorous exercise and time dedicated to sleep. The latter also correlated positively with the perception of health and academic performance and negatively with perception of fatigue, somatic symptoms, and systolic arterial pressure percentiles. When considering data from all the family members, we also observed interesting significant correlations between parents' exercise habits, parents' perceptions of health, and parents' perceptions of somatic symptoms and the perception of stress reported by their children, respectively.

**Conclusion:**

In this observational study, exercise and good sleep hygiene represent important tools to counteract stress perception in youth, fostering present and future wellbeing and health. The collection of lifestyle data using a simple questionnaire with simple clinical parameters may offer an opportunity to construct an immediate picture of family members' lifestyles, which may motivate parents and institutions to focus on improvement of lifestyle components (in particular, exercise and good sleep) instead of only focusing on traditional risk factors (such as dyslipidaemia, obesity, hypertension, and smoking) to foster present wellbeing in children/adolescents and prevent chronic non-communicable diseases.

## Introduction

A healthy lifestyle is currently considered a pillar in the prevention/treatment of chronic non-communicable diseases (CNCDs) in both adulthood and childhood (1–5). Notably, it is also a prominent tool for fostering wellbeing and managing stress, particularly at a young age when these two conditions play a fundamental role in determining present and future health (6–10).

Nevertheless, a lack of sufficient physical activity, poor nutrition, poor sleep, and smoking, are frequently present in children and adolescents (8, 9, 11, 12), who, despite these lifestyle factors, frequently present with clinical parameters, such as arterial pressure level, lipid profile, and plasma glucose levels (traditional cardiometabolic risk factors), within normal ranges, with the possible exclusion of an increased body mass index (BMI) (6). Campaigns to foster health and prevent chronic diseases often neglect some lifestyle components and focus mainly on the presence of specific cardiometabolic risk factors (8, 13, 14), such as overweight/obesity or dyslipidaemia, with a limited impact on youth behaviour, health, and wellbeing. Sedentariness, poor or insufficient sleep, unhealthy nutrition, and smoking may be unseen and/or considered “normal habits” as they are common to a large proportion of children and adolescents. Children's perceptions of their stress, health, and wellbeing are rarely assessed and considered.

Unfortunately, among children and adolescents, reduced wellbeing and increased stress are increasingly characteristic and represent a real issue (9–13), dramatically impacting present and future health, and even leading to growth alteration (15), psychiatric disorders (in particular anxiety and depression), and CNCDs in adulthood (8). Multiple mechanisms underlie this, ranging from physiological (autonomic, endocrine, and immunological controls) to psychological ones (16–18), and alterations in these control systems are responsible for the acute and/or chronic consequences of stressors. Nevertheless, stress also refers to an individual's subjective perception of stress (19) and one’s response to a stressor depends upon one’s individual characteristics. Moreover, one’s personal perception of stress plays a major role (20). Again, coping strategies, whether beneficial or detrimental, are also unique to an individual and are developed at a young age, possibly becoming lifelong (21). In this context, the adoption of a healthy lifestyle at a young age assumes great importance, both to prevent CNCD and to manage stress, and above all, to prevent the latter (11, 16, 22–25). In particular, being physically active and having good sleep have been shown to be useful in this regard (11, 22–28). Nevertheless, stressful conditions may impact physical activity habits and impair sleep (26, 27, 29–31). These considerations corroborate the need to promote healthy behaviours among children and adolescents and the importance of assessing lifestyle components in this population to detect any room for improvement as soon as possible. Thus, the role of parents needs to be accounted for. They may represent important role models (both positive or negative) and dramatically affect their children's wellbeing, potentially being a source of stress or support and joy.

The present study aimed to evaluate the link between stress perception and lifestyle, particularly exercise and sleep in children/adolescents and their parents, using a simple anonymous questionnaire. This study aimed to ascertain the prevalence of unhealthy lifestyles and, in particular, investigate the relationship between lifestyle components and stress perception, which may help parents and institutions to address this issue by intervening to manage/prevent stress. The easiest way to achieve this goal may be by improving their own and their children's behaviours.

## Methods

This observational study included data from 50 families (Table 1) and was conducted during an event to celebrate the 80th anniversary of CSI (Centro Sportivo Italiano), held in the Duomo Square of Milan in September 2024. In a section of the square, some gazebos dedicated to the promotion of health were set up. In particular, one of those was dedicated to the promotion of healthy lifestyles and its activities were managed by researchers from the Exercise Medicine Unit (Istituto Auxologico Italiano, University of Milan). All participants attending the event had the opportunity to receive an assessment of their lifestyle using a lifestyle questionnaire (see detailed description below), which was filled out with the assistance of a researcher who explained the meaning of each question (in particular, the questions regarding physical activity that considered the differences between exercise modalities and moderate–vigorous exercise vs. walking or other activities). Furthermore, the questionnaire collected simple clinical parameters (see detailed description below). After data collection, the researchers provided immediate feedback regarding each individual’s lifestyle profile and provided them with tailored counselling to improve their lifestyle. The participants spontaneously entered the gazebo, and when the researchers encountered a family (presence of a mother, a father, and one or more children together), they asked them to participate in the study and asked for permission to retain the anonymous collected data for research purposes. No other specific inclusion criteria (apart from being part of the family) were considered to respect privacy and anonymity. Age, anthropometric data (weight, height, and waist circumference), heart rate, and blood pressure were also assessed and the collected parameters were immediately noted in the questionnaire. All the data were gathered anonymously and the names of the single individuals were not collected and were never known by the researchers. No other personal data (address, sociodemographic data, job role, education, etc.) were collected to guarantee privacy and anonymity. The children's parents assisted their children in filling out the questionnaire and supervised the data collection. The participants were aware that the data would be used for research purposes. This research was part of a research protocol approved by the Istituto Auxologico Italiano IRCCS Ethic Committee (Istituto Auxologico Italiano, 18 April 2023, Code: 2023_04_18_14), and it was conducted following the Helsinki Declaration of 1975, as revised in 2008.

**Table 1:** Summary of the anthropometric and haemodynamic data from the study population.

Variable	Fathers	Mothers	Children
	Median ± MAD	Median ± MAD	Median ± MAD
N	36	45	73
Age (years)	50 ± 5	44 ± 5	12 ± 3
BMI (fathers and mothers: kg/m²; children: %)	26.02 ± 2.17	21.93 ± 2.56	51.00 ± 29
Weight (fathers and mothers: kg; children: %)	83 ± 6.5	59 ± 6.7	63.6 ± 20.8
WC (cm)	95 ± 9	78.5 ± 8.5	66 ± 7
Height (fathers and mothers: cm; children: %)	178.5 ± 3	165 ± 4	74.9 ± 20.15
SAP (fathers and mothers: mmHg; children: %)	120 ± 5	116 ± 5	85 ± 12
DAP (fathers and mothers: mmHg; children: %)	79 ± 5	77 ± 3	91 ± 8
HR (beats/min)	75 ± 7.5	75 ± 9	73.5 ± 7.5

BMI, body mass index; WC, waist circumference; SAP, systolic arterial pressure; DAP, diastolic arterial pressure; HR, heart rate.

Data are expressed as median value ± MAD.

An *ad hoc* part of the gazebo was dedicated to the clinical assessments. Weight was measured with the participants wearing light clothing without shoes. Height was assessed by researchers using a commercial statimeter, recording the participants’ heights with a precision of 0.5 cm. BMI was calculated as body weight (kg) divided by height (m^2^), and BMI percentile was calculated for children using the US Centers for Disease Control and Prevention’s (CDC) Child and Teen BMI Percentile Calculator (32).

We measured systolic (SAP) and diastolic arterial pressure (DAP) two to three times in the sitting position, using a manual sphygmomanometer (Certus, Tema, Milan, Italy) with an appropriately sized cuff on the right arm after 5 min of rest. We determined the blood pressure percentile for each child by following recent guidelines (33).

An *ad hoc* questionnaire was employed to quantify the following lifestyle components (34).

*Physical activity* (total activity volume) was assessed using a modified (Italian version) version of the commonly employed short version of the International Physical Activity Questionnaire (IPAQ) (35), which focuses on intensity [nominally estimated in metabolic equivalent of task (METs) according to the type of activity] and duration (in minutes) of physical activity. We decided to employ this questionnaire (36), even though it was designed for adults, because it has the advantage of providing a numeric parameter of exercise volume (expressed in METs), which reflects the total exercise volume. Moreover, we needed to quantify physical activity using the same instrument in all the enrolled participants. We considered the following levels: brisk walking (≈3.3 METs), other moderate-intensity activities (≈4.0 METs), and vigorous-intensity activities (≈8.0 METs) (35).

These levels were used to calculate the following:

– the total weekly exercise volume of exercise using the following formula: total weekly physical activity volume (METsTOT) [MET·min/week] = (3.3 × min of brisk walking × days of brisk walking) + (4.0 × min of other moderate-intensity activity × days of other moderate-intensity activities) + (8.0 × min of vigorous-intensity activity × days of vigorous-intensity activity).
– the total weekly exercise volume of moderate–vigorous exercise using the formula: METsMV = (4.0 × min of other moderate-intensity activity × days of other moderate-intensity activities) + (8.0 × min of vigorous-intensity activity × days of vigorous-intensity activity).

The IPAQ, as stated by previous researchers (35), has good reliability and validity values, as the “Typical IPAQ correlations were about 0.80 for reliability and 0.30 for validity, exhibiting measurement properties that were at least as good as other established self-report physical activity measures”.

*Nutrition* was assessed using the American Heart Association (AHA) Healthy Diet Score (37), adapted to Italian eating habits (34), considering fruit/vegetables, fish, sweetened beverages, whole grains, and sodium consumption. The AHA score uses integer values from 0 (“worst quality”) to 5 (“best quality”).

*Perceptions of stress, fatigue, and somatic symptoms* were assessed using a self-administered questionnaire (34, 38, 39), using nominal self-rated scales (higher values indicate higher degrees of symptoms) that focused on (i) the appraisal of one’s overall stress and fatigue perception by evaluation scales with integer scores from 0 (“no perception”) to 10 (“highest perception”) for each measure and (ii) the Short-Subjective Stress-related Somatic Symptoms Questionnaire (4S-Q), evaluating four somatic symptoms that account for the majority of somatic complaints. For scoring purposes, each response was coded from 0 (“no feeling”) to 10 (“a strong feeling”); thus, the total score ranged from 0 to 40.

The lifestyle questionnaire also inquired about the participants’ *hours of sleep/night and the individual's perception of their quality of health and their academic/job performance*. The latter were assessed using ordinal evaluation scales ranging from 0 (“worst quality”) to 10 (“best quality”).

### Statistical analysis

Descriptive statistics of the studied variables were computed as median ± MAD (median absolute deviation). One-way analysis of variance with Bonferroni *post hoc* analysis was performed to compare the variables among the father, mother, and children groups (non-parametric, independent-sample Kruskal–Wallis test). Spearman correlations were also employed. The statistical analysis was performed using SPSS version 29 (IBM Corp., Armonk, NY, USA). *P*-values < 0.05 were considered statistically significant.

## Results

Table 1 presents the participants’ ages, anthropometric data, and heart rate and blood pressure values. They were all in the normal range, but the fathers' BMI was slightly increased (26.02 kg/m^2^), indicating this group was slightly overweight.

Table 2 presents the lifestyle components. Notably, when considering the total weekly activity volume (METsTOT), all the participants met the World Health Organization’s guidelines on physical activity (adults reaching 600 METs/week and children/adolescents 1,200 METs/week) (1–4). When considering the total weekly exercise volume of moderate–vigorous exercise (METsMV), only the fathers and children/adolescents reached the goal.

**Table 2:** Summary of lifestyle and stress perception data from the study population.

Variable	Fathers	Mothers	Children
	Median ± MAD	Median ± MAD	Median ± MAD
METsTOT (kcal/min)	1,209.50 ± 912.5	678.00 ± 542.5	2,076 ± 960^b^
METsMV (kcal/min)	600 ± 600	240 ± 240	1,320 ± 920^a^^,^^b^
P. of somatic symptoms	5.50 ± 4	6 ± 5	5 ± 5
P. of stress	3 ± 3	5 ± 3	1 ± 1^a^^,^^b^
P. of fatigue	4 ± 2	5 ± 2	3 ± 2^a^^,^^b^
AHA.s	2 ± 1	2 ± 1	2 ± 1
Sleep hours (h/day)	7 ± 1	7 ± 1	8 ± 1^a^^,^^b^
P. of health	7 ± 1	7 ± 1	9 ± 1^a^^,^^b^
P. of performance	8 ± 1	8 ± 1	8 ± 1^b^

BMI, body mass index; WC, waist circumference; SAP, systolic arterial pressure; DAP, diastolic arterial pressure; HR, heart rate; METsTOT, total weekly physical activity volume; METsMV, weekly physical activity volume calculated only considering other moderate-intensity activities and vigorous-intensity activities (i.e., volume of moderate–vigorous exercise); P., perception; AHA.s, American Heart Association nutrition score; sleep.hours, self-reported number of hours per day of sleep.

Data are expressed as median value ± MAD.

^a^ *p* < 0.05 vs. fathers.

^b^ *p* < 0.05 vs. mothers.

The children/adolescents perceived their stress and fatigue to be significantly lower compared to their parents and they perceived their health and performance to be higher. Moreover, the children/adolescents slept for significantly longer, as expected.

**Figure 1:**
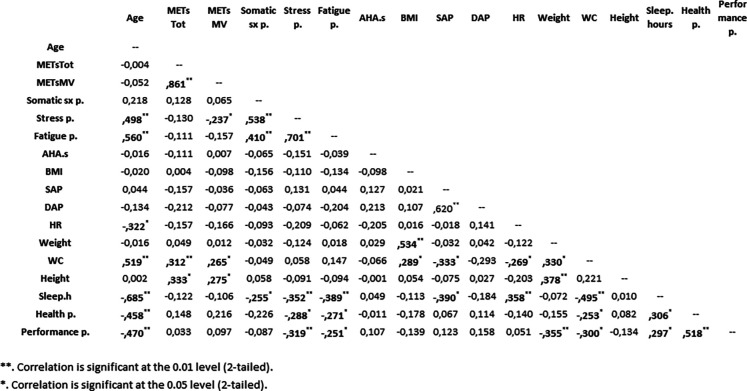
Simple correlation matrix of variables in the children/adolescents. METsTOT, total weekly physical activity volume; METsMV, weekly physical activity volume calculated only considering other moderate-intensity activities and vigorous-intensity activities (i.e., volume of moderate–vigorous exercise); somatic sx p., perception of somatic symptoms; stress p., perceptions of stress; fatigue p., perception of fatigue; AHA.s, American Heart Association nutrition score; BMI, body mass index; SAP, systolic arterial pressure; DAP, diastolic arterial pressure; HR, heart rate; WC, waist circumference; sleep.hours, self-reported number of hours of sleep per day; health p., perception of health; performance p., perception of performance. Significant values are in bold.

As shown in Figure 1, there were correlations (Spearman correlation analysis) between selected parameters. In particular, we observed that in the children/adolescents, the total weekly volume of moderate–vigorous exercise (METsMV) negatively correlated with stress perception. Interestingly, the number of sleep hours was negatively correlated with stress perception. Moreover, the number of sleep hours was also negatively correlated with perceptions of fatigue, somatic symptoms, health, and academic performance. Notably, the number of sleep hours was negatively correlated with systolic arterial pressure percentiles.

The mothers’ data revealed correlations between perception of health and stress (*r* = −0.28, *p* = 0.025), sleep hours (*r* = 0.27, *p* = 0.037), HR (*r* = −0.39, *p* = 0.003), and BMI (*r* = −0.38, *p* = 0.002). As expected, stress perception was significantly correlated with fatigue and somatic symptom perceptions (both *p* < 0.001).

The fathers’ data showed a correlation between METsMV and AHA score (a marker of nutrition quality) (*r* = 0.51, *p* = 0.001) and perception of health (*r* = 0.45, *p* = 0.001), which in turn correlated with perception of somatic symptoms (*r* = −0.43, *p* = 0.002), AHA score (*r* = 0.32, *p* = 0.022), BMI (*r* = −0.29, *p* = 0.038), and perceived performance (*r* = 0.43, *p* = 0.001). Moreover, the AHA score correlated with perception of fatigue (*r* = −0.32, *p* = 0.019). As expected, stress perception correlated with fatigue perception (*r* = −0.43, *p* = 0.001).

When considering correlations between data from all family members, we observed that METsTOT performed by mothers correlated both with METsTOT (*r* = 0.55, *p* < 0.001) and METsMV (*r* = 0.463, *p* = 0.002) performed by fathers. Furthermore, METsMV performed by mothers correlated with METsTOT (*r* = 0.38, *p* = 0.012) and METsMV (*r* = 0.465, *p* = 0.002) performed by fathers. Intriguingly, METsTOT performed by mothers correlated with fathers' age (*r* = −0.39, *p* = 0.011) and not with their own age. No significant correlation was observed between the exercise volume performed by parents and that of their children.

Perception of stress reported by the children/adolescents correlated with perception of somatic symptoms reported both by the mothers (*r* = 0.28, *p* = 0.023) and fathers (*r* = 0.30, *p* = 0.033).

Intriguingly, the fathers' sleep hours negatively correlated with both the mothers' perception of somatic symptoms (*r* = −0.40, *p* = 0.009) and fatigue (*r* = −0.42, *p* = 0.005), while the fathers' perception of health correlated with the mothers' perception of health (*r* = 0.73, *p* = 0.001)

## Discussion

The most interesting observation of this article is that, in children/adolescents, stress perception negatively correlates with the volume of moderate–vigorous exercise and time dedicated to sleep. The latter correlated positively with the children’s perceptions of health and academic performance and negatively with their perceptions of fatigue and somatic symptoms and the systolic arterial pressure percentiles. When considering data from all family members, we also observed interesting and significant correlations between the parents' exercise habits, perceptions of health, and perceptions of somatic symptoms, and the perception of stress reported by their children. We used a simple anonymous lifestyle questionnaire (34, 38–40) that provided data that allowed us to reveal the relationship between some lifestyle components and stress perception, and elucidate the important influence of the family environment on the family members' wellbeing.

One’s youth is the life period in which an individual shapes their future life and their present and future psychological/physical health. Youth is generally considered the happiest period of life; unfortunately, current evidence suggests that stress and reduced wellbeing are increasingly present among children and adolescents, representing an important health issue for families and institutions (9–13).

Stress is a multifactorial, complex phenomenon and can be defined in various ways. In general, it is defined as a state of threatened homeostasis with physical, psychological, and behavioural consequences, which are counteracted by adaptive processes in an attempt to regain homeostasis (18, 38, 41). An individual’s perception of stressful events is fundamental and perceived stress is defined as how stressful one appraises situations to be, which may include physical, psychological, actual, and anticipated aspects of stress (19). In this article, we assessed the perceptions of stress, fatigue, and stress-related somatic symptoms (42) using a self-administered questionnaire (34, 38–40) with nominal self-rated scales (higher values indicate higher degrees of perception) from 0 (“no perception”) to 10 (“highest perception”) for each measure. Our goal was to assess family members' perceived stress, focusing on their subjective experience rather than objective stressors (17), and to collect information related to more than one domain (43). This methodology may be simplistic; nevertheless, the scores correlate with physiological parameters such as markers of autonomic controls (13, 44, 45), and it is simple to use to study adults’ and children's perceptions of stress and/or stress-related somatic symptoms. In previous articles, we observed that, in young adults, stress perception was higher than in older adults (38), and it was greater in sedentary subjects compared to physically active subjects (38, 46). In this article, this simple method unveiled an important link between stress perception and reduced physical activity in children/adolescents and a link between stress perception and sleep.

Exercise and sleep represent important tools to minimize stress perception and, consequently, improve wellbeing, in addition to fostering future health. The evidence provided by this study may help parents move from a more traditional approach that considers only traditional specific cardiometabolic risk factors, such as overweight/obesity or dyslipidaemia, as determinants of their children's health to a proactive approach that improves their children’s lifestyle.

The importance of performing physical activity in early life is well-established, highlighting its role in the prevention/treatment of chronic disease and in the education of children/adolescents (1–4, 47). Less remarked on is its role in fostering wellbeing in all family members and improving performance. Indeed, it is a simple tool to consider.

The importance of sleep hygiene is growing, even among children/adolescents, as the last WHO guidelines for exercise in children (3, 4) indicated the amount of sleep and the volume of exercise required to improve health. Moreover, the literature showed a link between short sleep duration and elevated blood pressure in children (28, 48, 49), suggesting that sleep plays an important role in determining cardiovascular risk in youth.

In this study, we observed negative associations between sleep and perceptions of stress, fatigue, and somatic symptoms and blood pressure percentiles, and positive associations between sleep and health and academic performance. These findings highlight the importance of educating children/adolescents to get sufficient sleep, alongside exercising and mitigating sedentary behaviour.

Of particular interest in this study’s results is the association between the mothers' total and moderate–vigorous exercise and the fathers' total and moderate–vigorous exercise, respectively, suggesting the family’s important role in determining the behaviour of a single adult member. In our population, the fathers reported performing more moderate–vigorous exercise; however, the amount of moderate–vigorous exercise performed by the mothers increased as the exercise volume of the fathers increased, particularly if the fathers were younger.

The lack of correlation between the parents' and children's volumes of exercise in the studied population requires an explanation (50–52). A possible reason for this is that we collected data during an event dedicated to the promotion of sports that was organized by an institution specifically dedicated to promoting/organizing sports activities for youth and thus a large number of the children/adolescents participating in the event were likely to have participated in sports classes. Moreover, we were not able to collect data regarding the different sports disciplines, the actual exercise intensity performed, and the time spent in sedentary behaviour due to time constraints. We have to consider that, paradoxically, children/adolescents take part in sports activities rather than having active behaviour in everyday life. Studies show that parental lifestyle may affect children/adolescents' lifestyle behaviours (50–52); however, other factors may play a role, such as friends, social classes, and family finances (52). The family environment, furthermore, may dramatically affect sedentary behaviours. We have to specify that a decrease in sedentary behaviours is not synonymous with “performing structured exercise” but represents a specific attitude to take every opportunity to perform physical activity during everyday life. This attitude is independent of dedicating specific time to structured exercise and may be easier to maintain throughout life (53–55).

We should also highlight that increased exercise volume or increased pressure to perform at a high level in sports and poor sleep may represent sources of stress (27, 29–31). Family may also play a fundamental role in this regard. Parents should monitor their children’s/adolescents’ volume of exercise training and ensure they are not being pushed beyond their limits and are getting adequate sleep, even with the goal of performing in sport at a high level. This consideration may have particular value considering that the presence of stressors, which overcome the children's resources to manage them, may not only affect their wellbeing but also lead to worse lifestyle habits, such as smoking, unhealthy nutrition, and bad sleep hygiene.

## Limitations

We have to acknowledge some limitations of our study (16, 23, 29, 46). First, the study population was limited to 50 families who took part in an initiative to promote sport, limiting the generalizability of the observed results. Moreover, this relatively small population did not permit us to investigate some possible associations between different lifestyle parameters; for instance, the relationship between stress perception and exercise was not significant among the parents (45 mothers and 36 fathers), while it was significant among the 73 children/adolescents. We need to state that, although significant associations were identified in correlation analysis (*p* < 0.05), the small sample size may limit the generalizability of the results; future studies with larger samples are needed to validate these findings.

Second, we did not have any objective measure of exercise volume. Moreover, we are aware that the questionnaire we used to assess physical activity (35) has been validated for subjects older than 15 years, and that in our study population, there were younger subjects; nevertheless, for statistical reasons, we needed to quantify physical activity using the same instrument. Moreover, the employed questionnaire has the advantage of providing a numeric exercise volume parameter (expressed in METs), which reflects the participants’ exercise volume, while the questionnaire that has been validated for children did not have this feature (56). We have already used it in a paediatric population (36, 57), demonstrating its capability to provide data on exercise volume and its relationship with other objective physiological measures, such as autonomic, functional, and metabolic parameters, in both children (36, 57) and adults (38, 39, 44, 45). Obviously, this aspect represents an important limitation of our study, and further investigation will be necessary to confirm the data.

Third, we collected our data during an event to promote exercise, which may not be the ideal context for collecting physiological parameters such as blood pressure. We implemented all the precautions to adequately measure blood pressure, and the parents presented with blood pressure levels in the mean range of the healthy population, while the children/adolescents population was characterized by high mean blood pressure levels, although these were still within the normal range. However, it is possible that the children/adolescents participated in sports or activities during the event, enhancing sympathetic activity with obvious consequences for the blood pressure values.

## Conclusion

Exercise and good sleep hygiene represent important tools to counteract stress perception in youth, fostering present and future wellbeing and health. Family is a pivotal environment to instil healthy habits in children and adolescents, particularly performing physical exercise, reducing sedentary time, and promoting good sleep hygiene. The collection of lifestyle data using a simple questionnaire with simple clinical parameters during a social event that focused on fostering a healthy lifestyle provided an opportunity to construct an immediate picture of the family members' lifestyles and promote possible improvements, which are important for preventing chronic non-communicable diseases and fostering children/adolescents’ present wellbeing. These data are a valuable starting point for designing future studies in a large study population using the same validated questionnaire to assess the volume of physical activity in both children and adults. These studies are necessary to confirm the results presented in this study.

## Data Availability

The datasets presented in this article are not readily available because they contain sensitive information. Requests to access the datasets should be directed to daniela.lucini@unimi.it.
